# Literary Notes

**Published:** 1903-07

**Authors:** 


### ITEM.

The Maltine Company has published a very convenient and valuable chart of the principal poisons and their antidotes, which will be promptly sent to physicians and to hospitals, dispensaries, training schools for nurses and kindred institutions on application. Address, The Maltine Company, 8th Avenue and 13th Street, Brooklyn, N. Y.

